# Supplementary material for: Neural representation of goal direction in the monarch butterfly brain
Source: Nat Commun. 2023 Sep 20;14:5859. doi: 10.1038/s41467-023-41526-w (PMC10511513; doi:10.1038/s41467-023-41526-w)
Supplement: Supplementary file 1 — Supplementary Information [file 41467_2023_41526_MOESM1_ESM.pdf]

Title: Neural representation of goal direction in the monarch butterfly brain

M. Jerome Beetz [1]*, Christian Kraus[1,2], Basil el Jundi[1,2]

Affiliations

[1]Zoology II, Biocenter, University of Würzburg, Würzburg, Germany

[2]Animal Physiology, Department of Biology, Norwegian University of Science and Technology, Trondheim, Norway

*Corresponding author

M. Jerome Beetz: jerome.beetz@uni-wuerzburg.de

Keywords: navigation, orientation, migration, central complex, sun compass

Short title: Goal direction coding

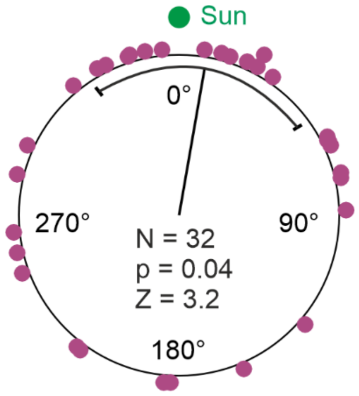

Figure S1. Distribution of mean headings relative to the virtual sun. Circular plot visualizing mean headings of 32 butterflies (magenta data points), sun positioned at 0°. Black line and sctor depicts the mean heading and 95% confidence intervals across the population of 32 butterflies. Statistics from a Rayleigh test, testing against a uniform distribution is depicted in the center of the circular plot. Source data file: datasource.xlsx Figure S1.

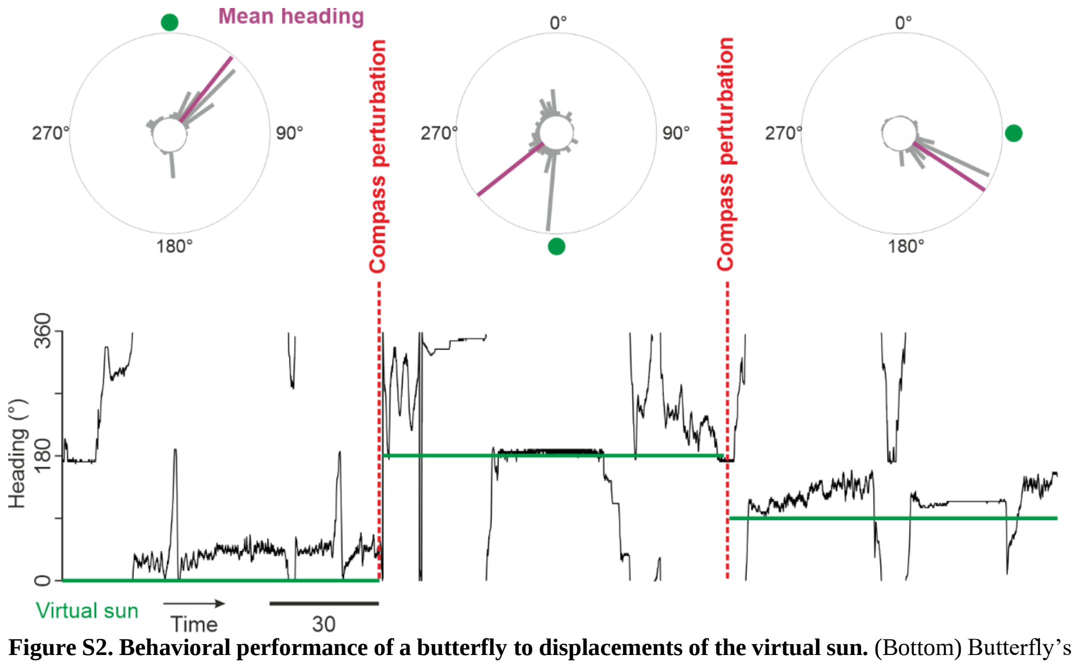

Figure S2. Behavioral performance of a butterfly to displacements of the virtual sun. (Bottom) Butterfly's heading (black trace) as a function of time. The angular position of the virtual sun is depicted in green. Every 90 seconds the virtual sun was displaced (compass perturbation). (Top) Circular histograms demonstrate the butterfly's heading (gray bars) at different virtual sun positions (depicted as green dots). Note that the butterfly changed its mean heading (magenta lines) to set a consistent goal heading relative to the virtual sun. Source data file: datasource.xlsx Figure S2.

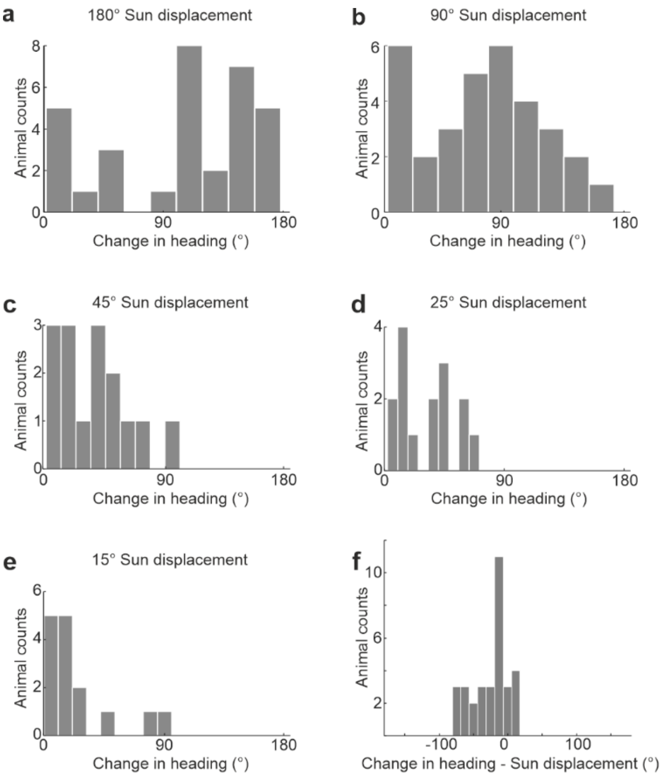

Figure S3. Summary of mean heading changes induced by displacing the virtual sun at different angular positions. Histograms showing the butterflies' change of heading after 180° (**a**), 90° (**b**), 45° (**c**), 25° (**d**), and 15° (**e**) sun displacements. (**f**) Angular difference between the change in heading and the sun displacement. Source data file: datasource.xlsx Figure S3.

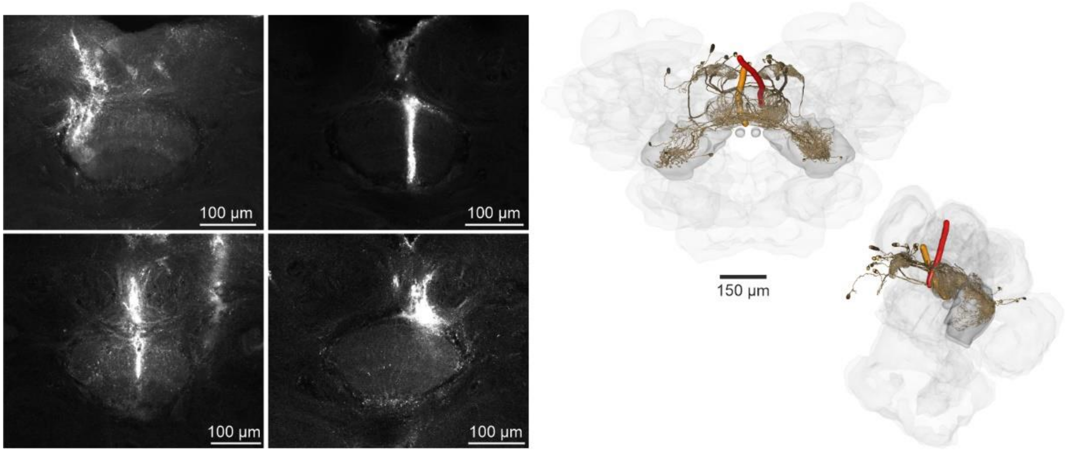

Figure S4. Visualiztion of tetrode positions in the butterfly brain. Left: Z-stacks from the fan-shaped body of the central complex showing stainings from four example tetrode tracks. Validation of the recording position in the central complex was successful for all 32 recorded butterflies. Right: Anterodorsal (left) and lateral (right) view of 3D reconstructed tetrode tracks that correspond to the example neurons presented in Fig. 1h and 2f-g. Prominent central-complex neurons (brown) are visualized. Source data file: datasource.xlsx Figure S4.

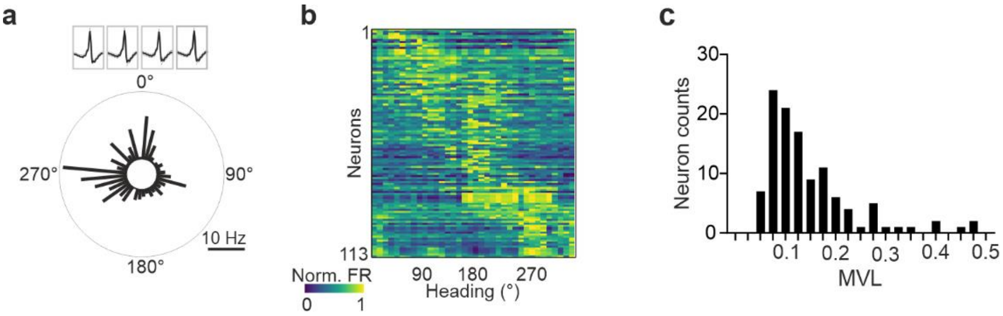

Figure S5. Central-complex neurons with an internal representation of directions. Angular tuning of an example (**a**) and 113 (**b**) neurons measured when the butterfly actively rotated on a platform in darkness. All neurons were spatially tuned according to a Rayleigh test ($p < 0.05$). Along y-axis, neurons are ordered according to their preferred firing directions. (**c**) Mean vector lengths (MVLs) of angular tuning from 113 neurons measured when the butterflies were orienting with respect to a virtual sun. Source data file: datasource.xlsx Figure S5.

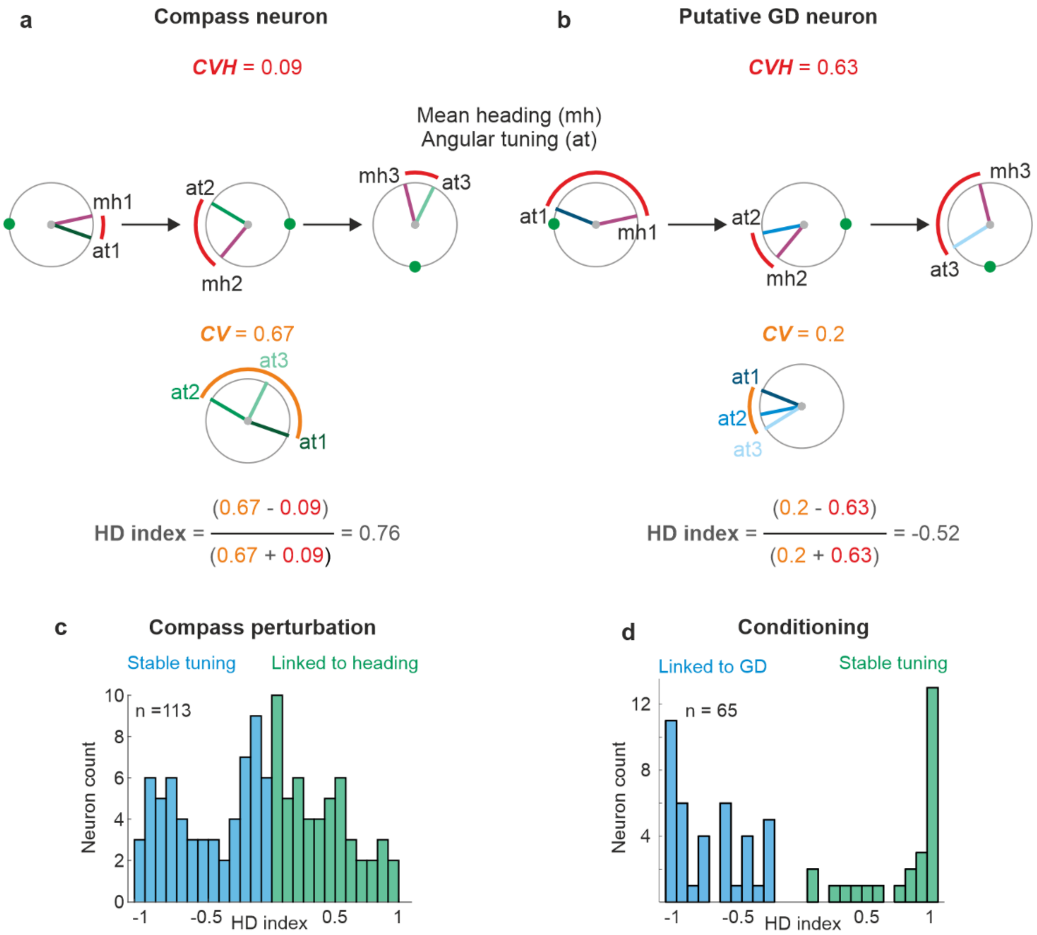

Figure S6. Categorization of neurons based on the HD index. (**a, b**) Schematic visualization of the HD index calculation used to categorize neurons into compass and GD neurons. A hypothetical angular tuning expected from a compass neuron (a) and a GD neuron (b) is shown. The circular variance of heading offsets (CVH) represents the variance of the angular relationship between the butterfly's mean heading (magenta bars) and the neurons' pfds across the compass perturbations (green for compass neuron and blue for GD neuron). The CVH is expected to be smaller for compass neurons than for GD neurons. As the butterfly's goal direction relative to the virtual sun was consistent in response to compass perturbations, angular tuning of GD neurons was expected to be invariant. The invariance of angular tuning was assessed by calculating the circular variance of pfds (CV) which was expected to be lower in GD neurons than in compass neurons. The HD index combines the neurons' CVH and CV. Neurons whose angular tuning can be best explained when considering the butterfly's heading direction have positive HD indices (a) while neurons with an invariant angular tuning may result in negative HD indices. (**c, d**) Histograms of measured HD indices from neurons recorded during compass perturbation (**c,** n = 113) and aversive conditioning (**d,** n = 65). Source data file: datasource.xlsx Figure S6.

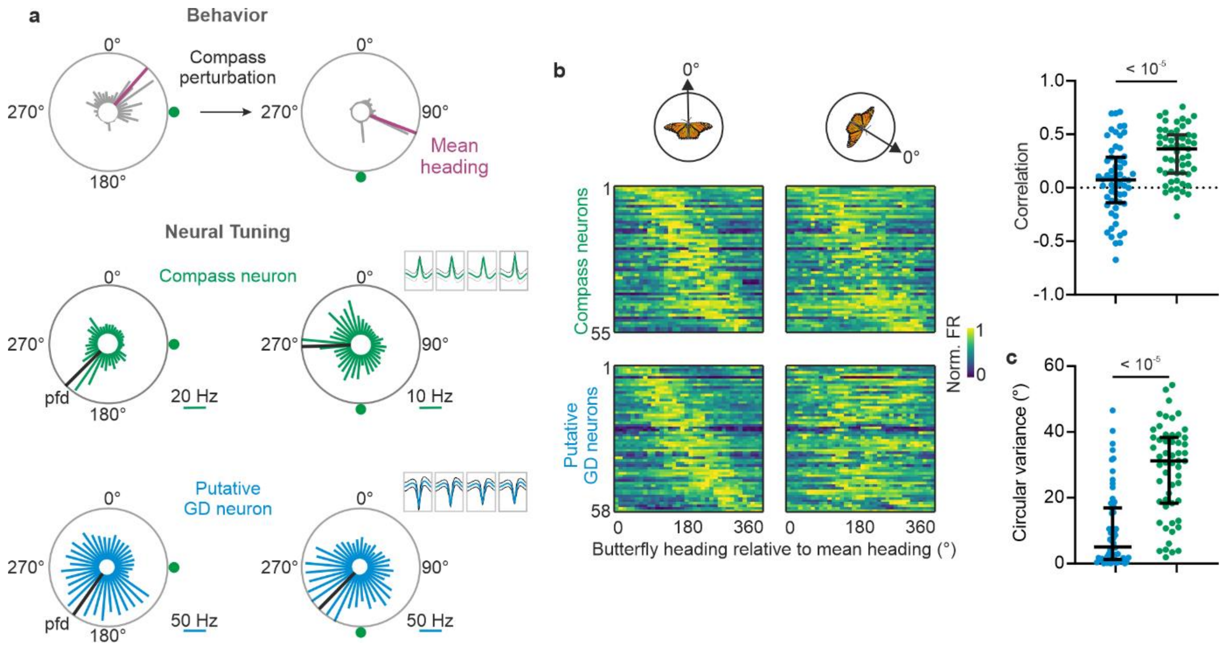

Figure S7. Discrimination of compass and putative GD neurons after sun displacements. (**a**) Behavioral response (upper circular plots) and neural tuning of two example neurons (green and blue) in response to a 90° sun displacement. Respectively, the mean heading and the preferred firing direction (pfd) are indicated by purple and black bars. The mean and percentile of the spike wave forms from each electrode is depicted in the right upper corner of the circular plots. Note that the angular tuning of the compass neuron follows the behavioral response and the pfd changes by about 90° while the angular tuning of the blue neuron is invariant. (**b**) left: Normalized firing rate of compass (n = 55) and putative GD neurons (n = 58) as a function of heading directions relative to the butterfly's body axis. Along y-axis, neurons are ordered according to their pfds before compass perturbation. Each neuron's firing rate (FR) is normalized against its peak firing rate. right: Correlation of the angular tuning referred to the butterfly's heading direction before and after compass perturbation. $p < 10^{-5}$, t = 4.442, df = 111, two-sided unpaired t test. (**c**) Comparison of circular variances of pfds in response to compass perturbations for putative GD (blue, n = 58) and compass (green, n = 55) neurons. $p < 10^{-5}$, U = 494, two-sided Mann-Whitney U. Error bars in b, and c represent the interquartile range with the median. Source data file: datasource.xlsx Figure S7.

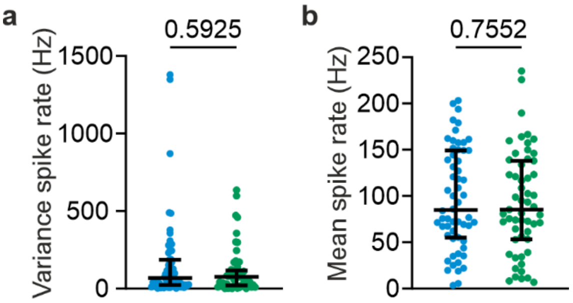

Figure S8. Overview of spike rate parameters for GD (blue) and compass neurons (green) during compass perturbations. (**a**) Mean spike rate for GD and compass neurons (two-sided Mann Whitney U test, variance: p = 0.7552, U = 1540, n = 55 compass neurons; n = 58 GD neurons). (**b**) Spike rate variance for GD and compass neurons (two-sided Mann Whitney U test, variance: p = 0.5925, U = 1501, n = 55 compass neurons; n = 58 GD neurons). Error bars represent the interquartile range with the median. Source data file: datasource.xlsx Figure S8.

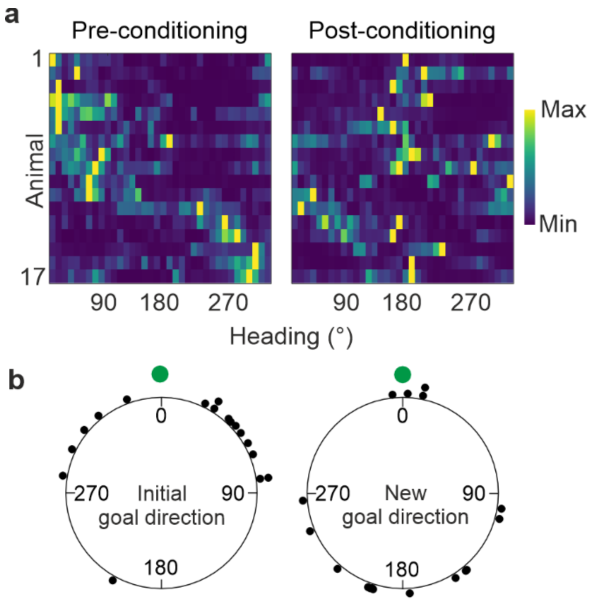

Figure S9. Behavioral performance of butterflies in response to aversive conditioning. (**a**) Distribution of normalized heading of 17 butterflies before (left) and after (right) conditioning. Headings were normalized against the peak heading for each butterfly. Along y-axis, butterflies were ordered according to their initial goal heading at pre-conditioning. (**b**) Circular plots of goal directions relative to the virtual sun (0°) prior to (left) and after conditioning (right). Source data file: datasource.xlsx Figure S9.

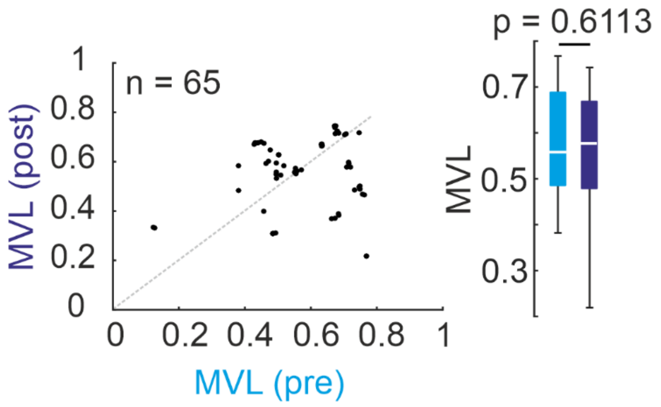

Fig. S10. Stable tuning directedness of central-complex neurons after aversive conditioning. Mean vector lengths from 65 central-complex neurons measured prior to (MVL pre, blue) and after conditioning (MVL post, purple) did not change in response to aversive conditioning (two-sided Wilcoxon matched-pairs signed rank test, $p = 0.6113$, $W = -157$, $n = 65$). Box plots indicate median (middle line), 25th, 75th percentile (box) and 5th and 95th percentile (whiskers). Source data file: datasource.xlsx Figure S10.

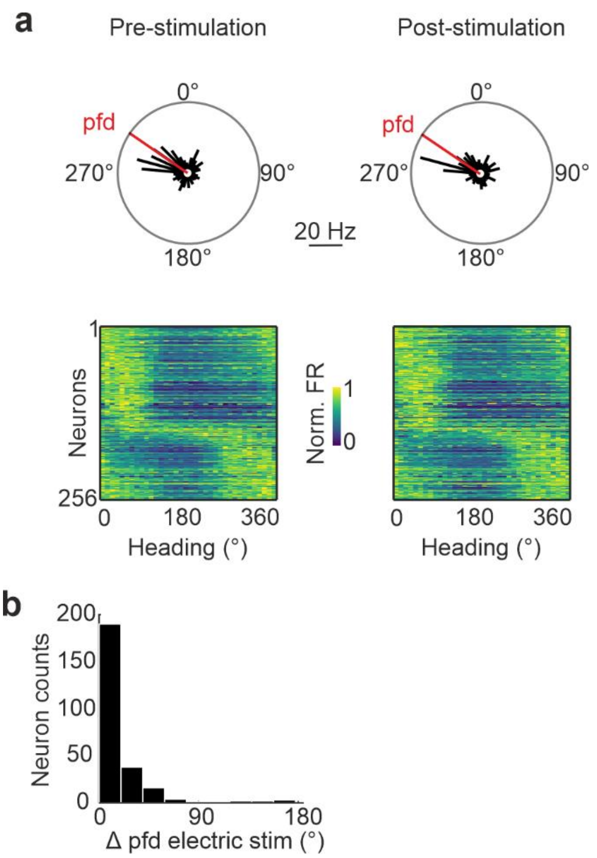

Figure S11. Electric stimulation in the central complex does not affect neural tuning. (**a**) Upper: Circular plots showing the angular tuning of a central-complex neuron measured with the virtual sun revolving around a restrained butterfly before (Pre-stimulation) and after (Post-stimulation) electric stimulation. Red line represents the neuron's preferred firing direction (pfd). Lower: Angular tuning of 256 central-complex neurons measured before and after electric stimulation. Each neuron's firing rate (FR) is normalized against its peak firing rate. Along y-axis, neurons are ordered according to their pfd. (**b**) Differences in pfds measured before and after electric stimulation. Electric stimulation does not change the neurons' pfds (two-sided Wilcoxon matched-pairs signed rank test, $p = 0.6323$, $W = 1136$, $n = 256$). Source data file: datasource.xlsx Figure S11.

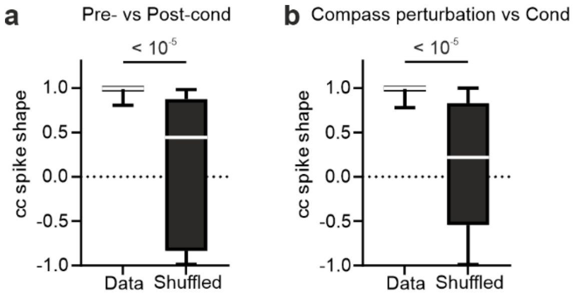

Figure S12. Spike shape stability of neurons recorded during the experiment. Correlation values (cc) of spike shapes measured before and after conditioning (**a**) and during compass perturbation and conditioning (**b**) were compared with correlation of spike shapes shuffled across randomly selected neurons [two-sided Wilcoxon matched-pairs signed rank test, $p < 10^{-5}$, W = -3395, n = 82; (a), $p < 10^{-5}$, W = -10276, n = 144 (b)]. Box plots indicate median (middle line), 25th, 75th percentile (box) and 5th and 95th percentile (whiskers). Source data file: datasource.xlsx Figure S12.

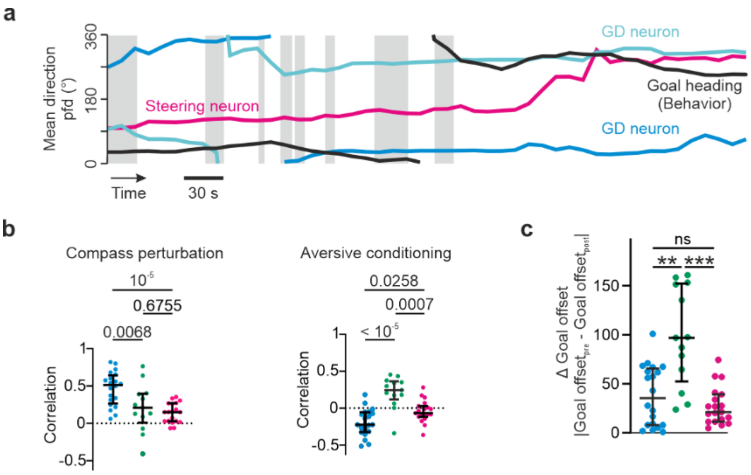

Figure S13. Angular tuning changes of GD, HD, and steering neurons in response to aversive conditioning. (**a**) Goal heading and pfds of example neurons (magenta: steering neuron, blue: GD neurons) plotted as a function of time (Time = 0 start of conditioning). Gray boxes highlight periods of electric stimulation. (**b**) Correlation of angular tuning before and after compass perturbations (left) or conditioning (right). In response to compass perturbation, only GD neurons (n = 20; blue) showed an invariant tuning indicated by significantly higher correlation values than for HD (n = 13; green) and steering neurons (n = 19; magenta) (ordinary one-way ANOVA: $p < 10^{-5}$, F = 11.07, n = 20 GD neurons, 13 HD neurons, 19 steering neurons; Tukey' s multiple comparison post-hoc correction test; HD vs. GD: p = 0.0068, q = 4.518; HD vs. steering: p = 0.6755, q = 1.199; GD vs. steering: $p < 10^{-5}$, q = 6.371). During aversive conditioning, angular tuning of HD neurons was invariant and the angular tuning of GD and steering neurons changed (ordinary one-way ANOVA: $p < 10^{-5}$, F = 20.72, n = 20 GD neurons, 13 HD neurons, 19 steering neurons; Tukey' s multiple comparison post-hoc correction test; HD vs. GD: $p < 10^{-5}$, q = 9.101; HD vs. steering: p = 0.0007, q = 5.62; GD vs. steering: p = 0.0258, q = 3.806). (**c**) Differences of goal offsets prior to and after conditioning. Note that angular tuning of GD (n = 20; blue) and steering neurons (n = 19; magenta) was dependent on the butterfly's goal direction while the angular tuning of HD neurons (n = 13; green) was independent (Kruskal Wallis test: p = 0.0002, KW statistic = 17.19, n = 20 GD neurons, 13 HD neurons, 19 steering neurons; Dunn' s multiple comparison post-hoc correction test; HD vs. GD: p = 0.0022 , Z = 3.377; HD vs. steering: p = 0.0002, Z = 3.949; GD vs. steering: p > 0.9999, q = 0.6813). Error bars in b, and c, represent the interquartile range with the median. Source data file: datasource.xlsx Figure S13.
