# Supplementary material for: Neural representation of goal direction in the monarch butterfly brain
Source: Nat Commun. 2023 Sep 20;14:5859. doi: 10.1038/s41467-023-41526-w (PMC10511513; doi:10.1038/s41467-023-41526-w)
Supplement: Supplementary file 3 — Description of Additional Supplementary Files [file 41467_2023_41526_MOESM3_ESM.pdf]

Description of Additional Supplementary Files

Supplementary Movie 1: Epoch of the butterfly's flight performance before conditioning.

Supplementary Movie 2: Epoch of the butterfly's flight performance after conditioning. Note that the butterfly tend to fly almost in the opposite direction to the direction prior to conditioning.
