# Supplementary material for: Neural representation of goal direction in the monarch butterfly brain
Source: Nat Commun. 2023 Sep 20;14:5859. doi: 10.1038/s41467-023-41526-w (PMC10511513; doi:10.1038/s41467-023-41526-w)
Supplement: Supplementary file 6 — Reporting Summary [file 41467_2023_41526_MOESM6_ESM.pdf]

Corresponding author(s): Jerome Beetz

Last updated by author(s): Aug 8, 2023

Reporting Summary

Nature Portfolio wishes to improve the reproducibility of the work that we publish. This form provides structure for consistency and transparency in reporting. For further information on Nature Portfolio policies, see our Editorial Policies and the Editorial Policy Checklist.

Statistics

For all statistical analyses, confirm that the following items are present in the figure legend, table legend, main text, or Methods section.

n/a	Confirmed	
☐	☒	The exact sample size (*n*) for each experimental group/condition, given as a discrete number and unit of measurement
☐	☒	A statement on whether measurements were taken from distinct samples or whether the same sample was measured repeatedly
☐	☒	The statistical test(s) used AND whether they are one- or two-sided<br>*Only common tests should be described solely by name; describe more complex techniques in the Methods section.*
☒	☐	A description of all covariates tested
☐	☒	A description of any assumptions or corrections, such as tests of normality and adjustment for multiple comparisons
☐	☒	A full description of the statistical parameters including central tendency (e.g. means) or other basic estimates (e.g. regression coefficient) AND variation (e.g. standard deviation) or associated estimates of uncertainty (e.g. confidence intervals)
☐	☒	For null hypothesis testing, the test statistic (e.g. *F*, *t*, *r*) with confidence intervals, effect sizes, degrees of freedom and *P* value noted<br>*Give P values as exact values whenever suitable.*
☒	☐	For Bayesian analysis, information on the choice of priors and Markov chain Monte Carlo settings
☒	☐	For hierarchical and complex designs, identification of the appropriate level for tests and full reporting of outcomes
☒	☐	Estimates of effect sizes (e.g. Cohen's *d*, Pearson's *r*), indicating how they were calculated

Our web collection on statistics for biologists contains articles on many of the points above.

Software and code

Policy information about availability of computer code

Data collection	Electrophysiological data were collected with the commercially available software Cheetah (version 6.4.2, Neuralynx Inc). Behavioral data were collected with the commercially available software US Digital. Imaging data were collected using the corresponding Leica software of the confocal laser scanning microscope TCS SP2. The LED arena was controlled with custom-written Arduino (version 1.8.15) scripts.
Data analysis	Spike2 (version 9.00, Cambridge Electronic Device) was used to spike sort the multichannel data. Custom-written Matlab (R2021a) scripts were used to analyze electrophysiological and behavioral data. Matlab and Arduino scripts can be accessed from the WueData database (https://doi.org/10.58160/92). For running the scripts, the following toolboxes/functions are required:<br>1. Circular Statistics toolbox by Behrens (https://de.mathworks.com/matlabcentral/fileexchange/10676-circular-statistics-toolbox-directional-statistics)<br>2. Customizeable Heat Maps by Deoras (https://de.mathworks.com/matlabcentral/fileexchange/24253-customizable-heat-maps)<br>3. Colormaps by Biguri (https://de.mathworks.com/matlabcentral/fileexchange/51986-perceptually-uniform-colormaps)<br>4. CircHist by Zittrell (https://de.mathworks.com/matlabcentral/fileexchange/66258-circhist-circular-polar-angle-histogram)<br>5. shadedErrorBar by Campbell (https://de.mathworks.com/matlabcentral/fileexchange/26311-raacampbell-shadederrorbar)<br>6. Statistics and Machine Learning Toolbox<br>7. Signal Processing and Communications Toolbox<br>Statistics were done in Oriana (version 4.01, Kovach Computing Systems) and GraphPad Prism 9 (GraphPad Software). The software Amira (version 5.3.3, ThermoFisher) was used to analyze imaging data.

For manuscripts utilizing custom algorithms or software that are central to the research but not yet described in published literature, software must be made available to editors and reviewers. We strongly encourage code deposition in a community repository (e.g. GitHub). See the Nature Portfolio guidelines for submitting code & software for further information.

Data

Policy information about availability of data

All manuscripts must include a data availability statement. This statement should provide the following information, where applicable:

- Accession codes, unique identifiers, or web links for publicly available datasets
- A description of any restrictions on data availability
- For clinical datasets or third party data, please ensure that the statement adheres to our policy

Neural raw data imported in Matlab and confocal scans of the Monarch butterfly brains are deposited at the WueData database https://doi.org/10.58160/92

Human research participants

Policy information about studies involving human research participants and Sex and Gender in Research.

Reporting on sex and gender	n/a
Population characteristics	n/a
Recruitment	n/a
Ethics oversight	n/a

Note that full information on the approval of the study protocol must also be provided in the manuscript.

Field-specific reporting

Please select the one below that is the best fit for your research. If you are not sure, read the appropriate sections before making your selection.

☒ Life sciences ☐ Behavioural & social sciences ☐ Ecological, evolutionary & environmental sciences

For a reference copy of the document with all sections, see nature.com/documents/nr-reporting-summary-flat.pdf

Life sciences study design

All studies must disclose on these points even when the disclosure is negative.

Sample size	We did not perform any statistics to determine the sample sizes prior to experiments. Sample size for the behavioral data was based on previous work [Franzke et al. (2020), Franzke et al. (2022)]. Sample size for electrophysiological recordings was based on Beetz et al. (2022).
Data exclusions	We did not exclude any animal from our experiments. Neurons that did not show a neural tuning in darkness (when the butterflies were walking on a platform) prior to flight where excluded, as a neural tuning in darkness is an important physiological feature of HD, steering and GD neurons.
Replication	All functionally described cell types, i.e., HD, GD and steering neurons have repeatedly been found in different butterflies and in different experiment trials conducted on different days. Behavioral responses to compass perturbations (N = 32) and aversive conditioning (N = 17) were highly robust across individual butterflies. For each experiment (N = 32), the recording position in the central complex has been confirmed anatomically.
Randomization	Degree of virtual sun displacement was presented in a random order to the animals. Our experimental design did not include sorting animals into control and experimental groups.
Blinding	As the nature of the applied electrophysiological technique does not allow any bias in the obtained neural recordings (they have been obtained blindly), no blinding was relevant during data acquisition. Similarly, as we were not able to monitor how the neurons changed their tuning when we manipulated the animals' behavior, no blinding was necessary. Classification in HD, steering, and GD neurons was not performed blindly, but was based on the outcome of the obtained experiments.

Reporting for specific materials, systems and methods

We require information from authors about some types of materials, experimental systems and methods used in many studies. Here, indicate whether each material, system or method listed is relevant to your study. If you are not sure if a list item applies to your research, read the appropriate section before selecting a response.

Materials & experimental systems

n/a	Involved in the study
☒	☐ Antibodies
☒	☐ Eukaryotic cell lines
☒	☐ Palaeontology and archaeology
☐	☒ Animals and other organisms
☒	☐ Clinical data
☒	☐ Dual use research of concern

Methods

n/a	Involved in the study
☒	☐ ChIP-seq
☒	☐ Flow cytometry
☒	☐ MRI-based neuroimaging

Animals and other research organisms

Policy information about studies involving animals; ARRIVE guidelines recommended for reporting animal research, and Sex and Gender in Research

Laboratory animals	Adult monarch butterflies of the species Danaus plexippus were used 1-2 weeks after eclosion. The butterflies were ordered as pupae from a commercial company (butterflyfarm.org, Costa Rica).
Wild animals	This study did not involve any wild animals.
Reporting on sex	We used female and male monarch butterflies in this study.
Field-collected samples	No field-collected samples were used in this study
Ethics oversight	Only invertebrates were used in this study, which do not require an animal protocol.

Note that full information on the approval of the study protocol must also be provided in the manuscript.
